# Accelerated 3D whole-brain T1, T2, and proton density mapping: feasibility for clinical glioma MR imaging

**DOI:** 10.1007/s00234-021-02703-0

**Published:** 2021-04-09

**Authors:** Carolin M. Pirkl, Laura Nunez-Gonzalez, Florian Kofler, Sebastian Endt, Lioba Grundl, Mohammad Golbabaee, Pedro A. Gómez, Matteo Cencini, Guido Buonincontri, Rolf F. Schulte, Marion Smits, Benedikt Wiestler, Bjoern H. Menze, Marion I. Menzel, Juan A. Hernandez-Tamames

**Affiliations:** 1 Department of Computer Science, Technical University of Munich, Munich, Germany; 2 GE Healthcare, Munich, Germany; 3 Radiology & Nuclear Medicine, Erasmus MC, University Medical Center, Rotterdam, Netherlands; 4 Department of Neuroradiology, Klinikum rechts der Isar, Munich, Germany; 5 Department of Computer Science, University of Bath, Bath, UK; 6 Fondazione Imago7, Pisa, Italy; 7 IRCCS Fondazione Stella Maris, Pisa, Italy; 8 TranslaTUM - Central Institute for Translational Cancer Research, Munich, Germany; 9 Department of Quantitative Biomedicine, University of Zurich, Zurich, Switzerland; 10 Department of Physics, Technical University of Munich, Munich, Germany

**Keywords:** MRI, Image-based biomarkers, Multiparametric imaging, Glioma imaging, Neural networks

## Abstract

**Purpose:**

Advanced MRI-based biomarkers offer comprehensive and quantitative information for the evaluation and characterization of brain tumors. In this study, we report initial clinical experience in routine glioma imaging with a novel, fully 3D multiparametric quantitative transient-state imaging (QTI) method for tissue characterization based on T1 and T2 values.

**Methods:**

To demonstrate the viability of the proposed 3D QTI technique, nine glioma patients (grade II–IV), with a variety of disease states and treatment histories, were included in this study. First, we investigated the feasibility of 3D QTI (6:25 min scan time) for its use in clinical routine imaging, focusing on image reconstruction, parameter estimation, and contrast-weighted image synthesis. Second, for an initial assessment of 3D QTI-based quantitative MR biomarkers, we performed a ROI-based analysis to characterize T1 and T2 components in tumor and peritumoral tissue.

**Results:**

The 3D acquisition combined with a compressed sensing reconstruction and neural network-based parameter inference produced parametric maps with high isotropic resolution (1.125 × 1.125 × 1.125 mm^3^ voxel size) and whole-brain coverage (22.5 × 22.5 × 22.5 cm^3^ FOV), enabling the synthesis of clinically relevant T1-weighted, T2-weighted, and FLAIR contrasts without any extra scan time. Our study revealed increased T1 and T2 values in tumor and peritumoral regions compared to contralateral white matter, good agreement with healthy volunteer data, and high inter-subject consistency.

**Conclusion:**

3D QTI demonstrated comprehensive tissue assessment of tumor substructures captured in T1 and T2 parameters. Aiming for fast acquisition of quantitative MR biomarkers, 3D QTI has potential to improve disease characterization in brain tumor patients under tight clinical time-constraints.

## Introduction

Gliomas are the most frequent primary brain tumors in adults. This diverse group of brain tumors comprises glioblastomas, astrocytomas, oligodendrogliomas, and ependymomas [1]. Although there have been great advances in glioma research, and treatment continues to evolve with new methods and strategies, gliomas remain a disease with poor prognosis [2]. State-of-the-art glioma treatment includes a multi-disciplinary approach, combining surgical resection, chemotherapy, and radiation therapy [3]. Treatment strategy and prognosis for each individual case depend on tumor grade, which is defined upon histopathologic appearance and molecular features according to the 2016 WHO criteria [4]. High-grade gliomas (grade IV), so called glioblastomas, are aggressive, fast-growing tumor types that require immediate treatment. For lower-grade gliomas (grades II, III), including various types of astrocytic, oligodendroglial, and ependymal tumors, extensive treatment is often delayed as long as possible [5].

For all types of gliomas, comprehensive multimodal neuroimaging is fundamental for disease characterization [6, 7]. It also guides the individualized therapy planning and is required to monitor treatment response and progression of the disease. Here, MRI has become the key diagnostic measure for the evaluation and characterization of brain tumors: while the multitude of image contrasts of conventional structural MRI allows for better detection of tumor-infiltrated areas, advanced image-based physiologic and molecular biomarkers have been demonstrated to offer comprehensive and quantitative information about the biological characteristics of tumor types and tumor substructures [8]. For the ultimate goal of an as-precise-as-possible therapy [9], quantitative MRI can therefore provide versatile tissue characterization [10]. This in turn is essential to better comprehend the complex proliferative and invasive behavior, to identify and describe structures of interest, such as enhancing tumor structures, or critical thresholds in a reliable and reproducible way to better predict therapy response and treatment outcomes.

Usually, long acquisition times of such conventional quantitative MR techniques, however, hinder their adoption into clinical practice. Routine imaging protocols therefore rely on mainly qualitative information so far. Also, visual inspection and qualitative interpretation are dominating clinical MRI-based diagnosis because the analysis of complex multi-parametric, multimodal, and even multi-temporal image data sets remains a major challenge. These issues, together with the lack of MRI protocol standardization [11], hamper a reliable identification of tumor substructures, render an exact quantification of infiltration patterns impossible, and complicate monitoring of treatment response in follow-up examinations.

To meet the clinical need for fast acquisition of quantitative MR biomarkers, advanced multiparametric MRI schemes have been proposed, offering reproducible and accurate diagnostic information, which is less affected by system and interpretation biases [12–16]. They all share the common goal of revealing clinically relevant tissue characteristics, which are not appropriately captured in standard qualitative MRI, with clinically practicable scan times.

Aiming for joint T1 and T2 mapping, different acquisition and data processing strategies based on (undersampled) k-space data are used to achieve optimal multiparametric estimation [12–15, 17]. In this work, we present the feasibility of a novel, fully 3D multiparametric quantitative transient-state imaging (QTI) technique [18] for simultaneous mapping of T1 and T2 relaxation times and relative proton density (PD) for use in clinical routine glioma imaging. With 3D QTI, we demonstrate a 3D acquisition with high isotropic resolution that allows us to go beyond the resolution of other recently presented quantitative MRI methods based on 2D (multi-)slice acquisitions. We pursue a conceptionally different approach compared to steady-state magnetization techniques and acquire the signal evolution in the transient state. In contrast to Cartesian readout schemes, the combination of efficient spiral k-space (under-)sampling with transient-state imaging in 3D QTI constitutes an attractive candidate for fast multiparametric MRI under tight clinical time constraints.

As a form of clinical stress test for 3D QTI, we chose a variety of glioma patients (grades II–IV) with heterogenous disease states and treatment histories to demonstrate the viability of this technique. In this study, we focus on two main aspects:

I. Initial clinical experience with 3D QTI: We demonstrate the feasibility of this fast, multiparametric sequence with whole-brain coverage and high isotropic resolution for being used in routine brain tumor imaging protocols. We examine the applicability of 3D QTI with its clinically relevant scan time of 6:25 min, focusing on its image reconstruction and parameter estimation approaches. Based on the quantitative parameter maps, we synthesize qualitative image contrasts and explore their clinical relevance. We also assess the behavior of the 3D QTI scheme in the presence of patient movement, i.e., rigid head motion and non-rigid physiological motion.
II. Application to quantitative characterization of tumor substructures: We identify tumor tissue heterogeneity that is captured by T1 and T2 values to offer comprehensive tissue assessment of tumor substructures and quantifiable differentiation of healthy tissue. We therefore characterize T1 and T2 components in a variety of glioma patients with different disease stages and treatment histories. We aim to gain insights into potential benefits of 3D QTI in cases where pseudo-regression, pseudo-therapy response, or radiation-induced necrosis complicate follow-up assessments [19].

## Materials and methods

### Subjects

Within the course of this study, we collected MR data and respective demographic and clinical data from nine glioma patients who had been scheduled for follow-up clinical imaging (Table 1). The study included a variety of patients who were initially diagnosed with glioblastoma (anaplastic and low-grade), astrocytoma (transitional cell and anaplastic), or oligodendroglioma. Prior treatment strategies cover surgical resection, chemotherapy, radiation therapy, or a combination thereof.

**Table 1:** Patient demographics, diagnoses, and treatment histories

Patient ID	Age	Gender	Diagnosis	Treatment
1	69 y	f	Giant cell glioblastoma, IDH wild type	Resection, radiation therapy, chemotherapy
2	63 y	m	Anaplastic astrocytoma (WHO grade III)	Chemotherapy
3	49 y	m	Glioblastoma, IDH wild type	Chemotherapy
4	69 y	m	Glioblastoma	Resection, chemotherapy
5	63 y	m	Transitional cell oligodendroglioma (WHO grade II)	Resection, radiation therapy
6	52 y	f	Glioblastoma	Resection, radiation therapy, chemotherapy
7	50 y	m	Oligodendroglioma (WHO grade II)	Resection, radiation therapy, chemotherapy
8	58 y	f	Anaplastic oligodendroglioma, IDH mutant and 1p/19q co-deleted	Resection, chemotherapy
9	25 y	f	Low-grade astrocytoma	Resection, chemotherapy

### MR imaging

#### Clinical contrast-weighted MRI

All MRI data were acquired on a 3T MR750 system (GE Healthcare, Milwaukee, WI) using a 16-channel head, neck, and spine array coil. The multimodal MRI protocol included a pre-contrast T1-weighted fast spoiled gradient echo (FSPGR) sequence (T1w), a T2-weighted PROPELLER sequence (T2w), and a fluid-attenuated inversion recovery (FLAIR) sequence which were followed by a gadolinium (Gd)-enhanced T1-weighted FSPGR sequence (T1c). All imaging parameters are shown in Table 2.

**Table 2:** MR sequence parameters

	3D QTI	T1-weighted FSPGR (T1w)	Gd-enhanced T1-weighted FSPGR (T1c)	T2-weighted PROPELLER (T2w)	CUBE FLAIR (FLAIR)
Acquisition	3D	3D	3D	2D	3D
Native resolution (mm^3^)	1.125 × 1.125 × 1.125	0.47 × 0.47 × 0.8	0.47 × 0.47 × 0.8	0.5 × 0.5 × 3.3	0.8 × 0.47 × 0.47
Matrix size	200 × 200 × 200	512 × 512 × 212	512 × 512 × 212	512 × 512 × 46	192 × 512 × 512
Field of view (mm^3^)	225 × 225 × 225	240 × 240 × 170	240 × 240 × 170	260 × 260 × 152	154 × 240 × 240
Slices	-	-	-	46	-
Native slice thickness (mm)	-	-	-	3.0	-
TE (ms)	1.8	2.1	2.1	120.7	92
TR (ms)	7.8	4.6	7.1	5751	5002
TI (ms)	18	-	-	-	1701
*ɑ* (°)	0.8 ≤ *ɑ* ≤ 70	12	12	160	90
Acquisition time (min)	6:25	1:54	4:43	5:15	4:48

#### 3D QTI acquisition and reconstruction

In addition to the clinical sequences, and before contrast agent administration, the patients were scanned with the proposed 3D QTI acquisition with an inversion time TI = 18 ms, repetition time TR = 7.8 ms, and echo time TE = 1.8 ms. Flip angles (0.8° ≤ *ɑ* ≤ 70°) follow a ramp-up/ramp-down pattern, comprising 880 repetitions. Highly undersampled k-space data (undersampling factor of 628 for each of the 880 3D k-space volumes) is acquired in the transient state [16, 20] using a spiral readout (22.5 × 22.5 × 22.5 cm^3^ FOV, 1.125 × 1.125 × 1.125 mm^3^ isotropic voxel size) with in-plane and spherical rotations to achieve full 3D coverage. The total scan time of the 3D QTI acquisition was 6:25 min.

**Fig. 1:**
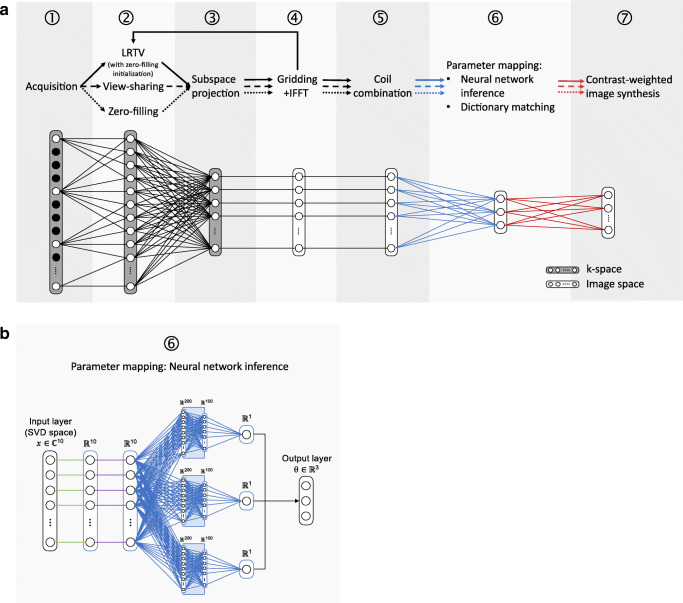
3D QTI data processing. **a** Reconstruction and processing. After acquisition (➀), raw k-space data is processed via naïve zero-filling (dotted line), k-space weighted view-sharing (dashed line), or a compressed sensing LRTV technique (solid line). All methods in ➁ are followed by dimensionality reduction via SVD subspace projection in the time domain (➂), gridding onto a Cartesian grid followed by a 3D IFFT (➃), and coil sensitivity estimation and combination (➄). The reconstructed image series are then fed into a neural network or are matched to a precomputed dictionary to output parametric maps of T1, T2 and PD (➅). We then synthesize clinical image contrasts using the parametric maps (➆). **b** Neural network architecture for parameter inference. The model receives the complex, voxel-wise signal in SVD subspace *x* and infers the underlying tissue parameter vector ***θ*** with T1, T2, and a PD-related scaling factor. The input signal ***x*** is phase-aligned (green lines) to transfer the complex into real-valued signal, followed by a normalization layer (purple lines). The model then divides into separate pathways, each with three ReLU-activated hidden layers and 200, 100, and one node, to eventually yield the concatenated parametric output vector ***θ***

3D QTI data was reconstructed using a compressed sensing (CS) approach with joint spatial and temporal regularizations, referred to as low-rank and total-variation (LRTV) method [21]. To demonstrate the anti-aliasing that is achieved by this iterative k-space processing, we compared this reconstruction to naïve zero-filling and k-space weighted view-sharing [22]. Figure 1 a schematically shows the 3D QTI reconstruction pipeline with these three k-space processing alternatives in step ➁. In all cases, we applied dimensionality reduction via SVD subspace projection (step ➂) to compress the full temporal signal evolution to its first ten singular images. SVD projection was followed by gridding onto a Cartesian grid using gpuNUFFT [23] and 3D inverse fast Fourier transform (IFFT, step ➃). In step ➄, coil sensitivity maps were computed using adaptive coil combination [24].

For parameter mapping in step ➅, the reconstructed, complex SVD images were fed into a compact multi-path neural network for voxel-wise T1, T2, and PD inference, which has shown to be a time and memory-efficient alternative to conventional dictionary matching [25, 26]. Note that neural network inference and dictionary matching can be applied independent of the previously performed k-space processing.

The proposed neural network architecture, as depicted in Fig. 1b, receives the first ten singular components of the SVD compressed QTI signal **x** as input and outputs the underlying tissue parameters T1, T2, and a PD-related scaling factor, comprised in the output vector **θ**, with the final PD estimate $\mathrm{PD}=\frac{{\left\Vert \mathbf{x}\right\Vert}_2}{\theta_3}.$ The latent-space input signal is phase-aligned [18], transferring the complex into a real-valued signal, and normalized in the subsequent layer. The model then splits into separate pathways, each consisting of three hidden layers with rectified linear unit (ReLU) activations and 200, 100, and one node, to eventually form the concatenated parametric output **θ**.

To train the neural network, we generated a dataset of synthetic QTI signals for 10 ms ≤ T1 ≤ 5000 ms and 10 ms ≤ T2 ≤ 2000 ms using the extended phase graphs formalism [27]. T1 values were sampled in steps of 10 ms for 10 ms ≤ T1 ≤ 2000 ms and in steps of 100 ms for 2100 ms ≤ T1 ≤ 5000 ms. T2 values were increased in steps of 5 ms for 10 ms ≤ T2 ≤ 300 ms and in steps of 10 ms for 310 ms ≤ T2 ≤ 2000 ms. The dataset was also used to obtain a dictionary matching reference. For model training, we used 80% of the samples in the simulated dataset and added white complex Gaussian noise to the generated signal time-series. The network was trained for a maximum of 1000 epochs with mean absolute percentage error loss and stochastic gradient descent optimization with a learning rate of 1e-4 and a dropout rate of 0.8. We kept the model state that achieved the best validation loss for the remaining 20% of the signals.

With the obtained T1, T2, and PD estimates, we generated synthetic T1-weighted, T2-weighted, and T2-weighted FLAIR image contrasts by applying the respective voxel-wise signal equations to the estimated parameter maps, as motivated by [18, 28]. To assess the quality of the synthetic images, we evaluated them against the corresponding acquisitions in the clinical protocol.

### Annotation and quantitative analysis of tumor substructures

**Fig. 2:**
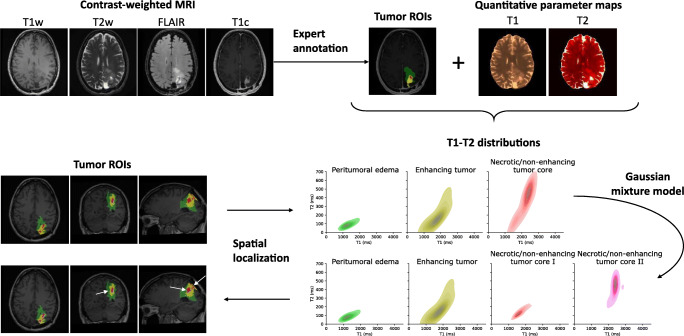
Application to quantitative characterization of tumor substructures. For quantitative analysis of tumor substructures, intra-tumoral structures, i.e., peritumoral edema, necrotic/non-enhancing tumor core, and enhancing tumor, were annotated by a trained expert based on the clinical contrast-weighted MR data. Voxel-wise T1-T2 distributions were then derived for the individual ROIs. Using a Gaussian mixture model, we explored whether we can identify the two voxel classes that are apparent in the T1-T2 space in necrotic/non-enhancing tumor areas, which were then mapped back to the image space

For quantitative analysis of tumor substructures in terms of T1 and T2 parameter values, as schematically shown in Fig. 2, intra-tumoral structures—peritumoral edema, necrotic/non-enhancing tumor core, and enhancing tumor core—were annotated by a trained radiologist using ITK SNAP [29] in each patient dataset based on the clinical contrast-weighted MRI data. The T1-weighted, T2-weighted, FLAIR, and Gd-enhanced T1-weighted images were therefore transformed into the 3D QTI image space using ANTs [30].

In addition to the tumor annotations, we obtained white matter (WM) and gray matter (GM) tissue segmentations using the FSL FAST algorithm [31], which we applied to the synthesized T1-weighted image data. For the glioma patients, we only considered contralateral WM and GM. If the tumor or its peritumoral tissue affected both hemispheres, normal-appearing WM and GM regions were delineated in the hemisphere with less tumor-affected volume.

Voxel-wise T1-T2 distributions were then identified for individual ROIs. We also compared ROI-based mean T1 and T2 among individual patients. For an initial attempt to explore whether the obtained T1 and T2 information allows us to go beyond the manual segmentation, we fitted a Gaussian mixture model to the T1-T2 space of all necrotic/non-enhancing voxels in the cohort to understand whether we can identify the two apparent tissue types, which we attribute to solid tumor (necrotic/non-enhancing tumor core I) and fluidic tissue voxels (necrotic/non-enhancing tumor core II) therein. The fitted model was then applied to the individual patient datasets to disentangle the voxels in the necrotic/non-enhancing ROI into two classes. The thereby obtained subclassification of necrotic/non-enhancing voxels was then mapped back to the anatomical context to complement the clinical baseline labeling based on qualitative visual MRI contrasts

## Results

We first present and evaluate the 3D QTI method with its modular data processing pipeline. Accuracy and precision of acquisition and reconstruction elements of 3D QTI were evaluated in [18, 21, 32]. Focus of the work presented here is to assess the applicability of 3D QTI for clinical routine imaging in terms of reconstruction performance and image quality of the multiparametric maps. We then use the multiparametric output of 3D QTI to identify tumor tissue heterogeneity and to understand whether this allows a quantifiable differentiation of tumor substructures and healthy tissue in cancer patients.

### Initial clinical experience with 3D QTI

**Fig. 3:**
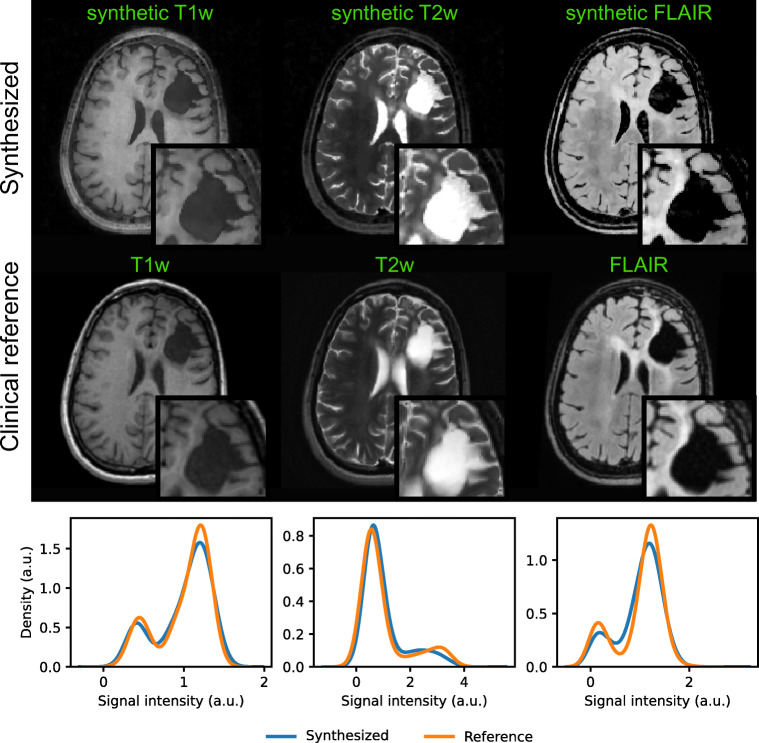
Contrast-weighted image synthesis for a representative patient case. From the T1, T2, and PD maps, we produce clinically relevant, fully 3D qualitative image information with high isotropic resolution and without additional scan time. As seen from the axial views and the histogram-based comparison considering the whole image volumes, synthetic T1-weighted, T2-weighted, and FLAIR MRI contrasts correspond to the clinical reference acquisitions. Corresponding sagittal and coronal views are shown in Fig. 11

**Fig. 4:**
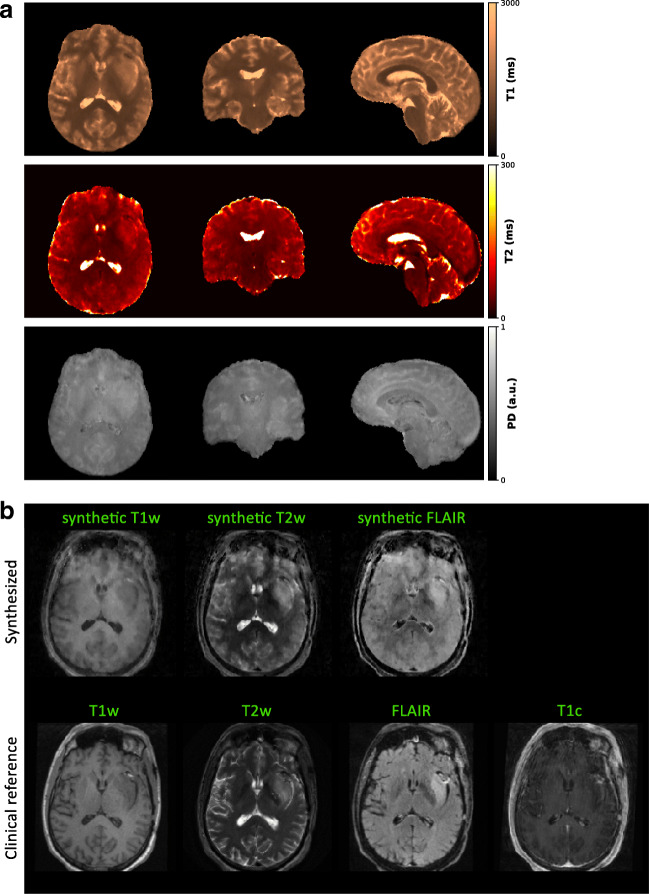
Sensitivity to rigid head motion. Profuse head motion can affect image acquisition in the transient-state, which leads to image degradation in the parametric maps (**a**) and the synthetic image contrasts (**b**) compared to the clinical contrast-weighted acquisitions. The post-contrast T1-weighted MRI indicates that state-of-the-art conventional MRI cannot fully recoup the pronounced head motion in this case. Corresponding sagittal and coronal views are shown in Fig. 12

**Fig. 5:**
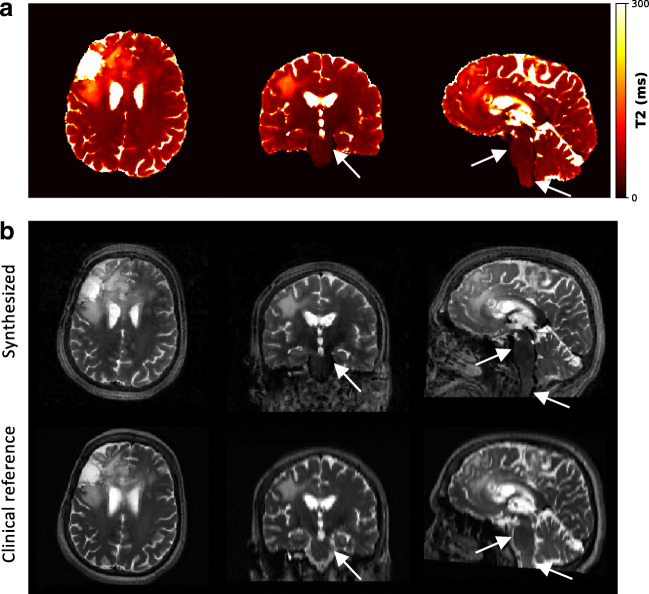
Sensitivity to physiological motion. Pulsating blood flow and thereby induced pulsation of the cerebrospinal fluid (CSF) can impact the T2 estimation (**a**) and subsequent synthesis of T2-weighted image contrasts (**b**) as observed in large vessels and in regions with high CSF pulsation, e.g., along the brainstem (white arrows)

Figure 9 illustrates the isotropic 3D maps of T1, T2, and PD that we obtained from the three reconstruction modules with subsequent dictionary matching for a representative patient case and a healthy volunteer. All subsequent results in this study rely on LRTV-based image reconstruction. Figure 10 shows parameter quantification results obtained via neural network-based inference and conventional dictionary matching for a representative patient dataset and a healthy volunteer. From the estimated T1, T2, and PD maps, we synthesized common MRI contrasts using the respective MR signal equations. In Fig. 3 and Fig. 11, we compare the synthetic images for T1-weighted, T2-weighted, and FLAIR image contrasts to the images that were acquired as part of the clinical protocols. As part of the sensitivity analysis towards rigid head movements and physiological motion, Fig. 4 and Fig. 12 show an exemplary case of pronounced patient movement, where motion-related artifacts degrade the image quality in the parametric maps and therefore affect the synthesized MRI contrasts. From Fig. 5 and Fig. 12, we observe how physiological motion, such as blood flow and CSF pulsation effects, impact parameter quantification and subsequent contrast-weighted image synthesis.

### Application to quantitative characterization of tumor substructures

**Fig. 6:**
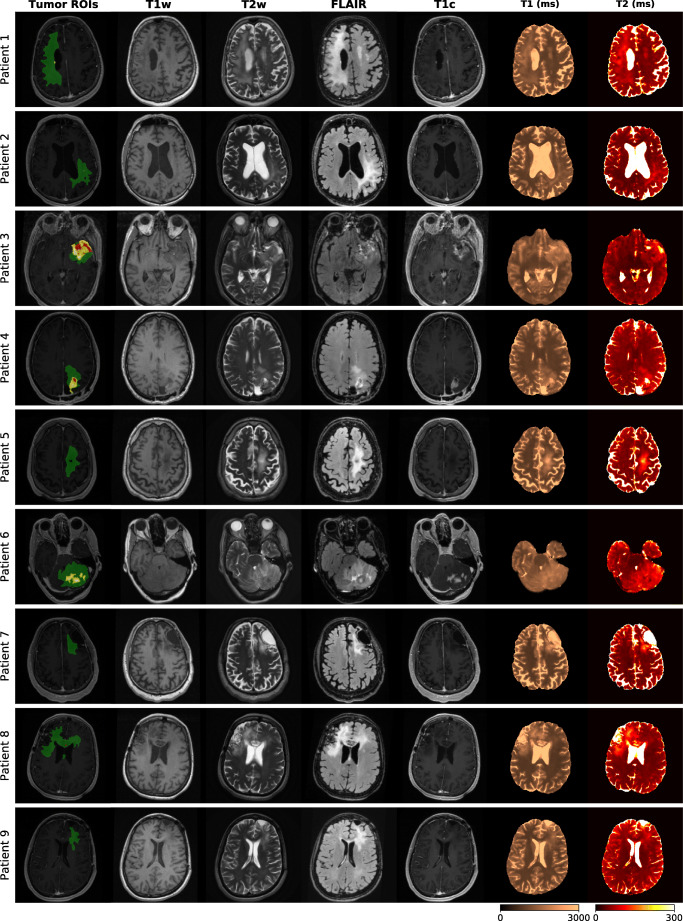
Qualitative comparison of tumor patient cases. Expert ROIs (green: peritumoral edema, red: necrotic core/non-enhancing tumor, yellow: enhancing tumor) are shown together with clinical T1-weighted FSPGR, T2-weighted, FLAIR, Gd-enhanced T1-weighted FSPGR images and quantitative T1 and T2 maps

Figure 6 gives an overview of the tumor ROI annotations together with the clinical contrast-weighted MRIs and the obtained T1 and T2 maps for all patients. In all cases, tumor core (red annotation) and peritumoral edema regions (green annotation) appear hyperintense on the conventional T2-weighted and FLAIR images and hypointense on T1-weighted images, relative to normal appearing tissue. T1 and T2 values obtained in these regions are higher compared to healthy tissue areas. Post-contrast T1-weighted images of patients 1 to 4 and 6 additionally identify areas with Gd enhancement (yellow annotation). Patients 3, 4, and 6 are cases with clearly visible gross tumor volumes. For patients 1 and 8, contrast-weighted MRIs do not indicate tumor relapse around the resection cavities. In case of patient 8, there are small tumor-suspected findings in the corpus callosum and the left anterior horn of the lateral ventricle without hyperintensities in the post-contrast T1-weighted MRI. In case of patient 1, the post-contrast MRI reveals small findings that are positive for Gd enhancement. For patient 2, there are small, discrete areas of Gd enhancement, which might indicate diffuse tumor growth, surrounded by edema. For patient case 5, 7, and 9, there is no clear sign for tumor reoccurrence after resection. In these cases, tumor ROIs only comprise areas of peritumoral edema and gliosis.

**Fig. 7:**
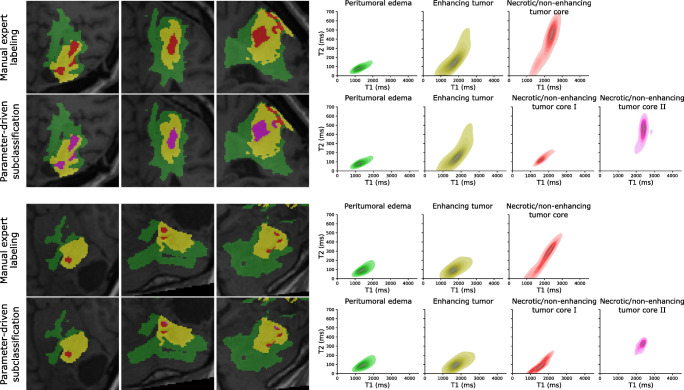
Qualitative T1-T2-analysis based on manual ROI annotations together with additional explorative parameter-driven tumor subclassification for two representative patient cases. Classification of necrotic/non-enhancing tissue voxels based on quantitative T1 and T2 values can give more insights into the heterogenous structure, which we attribute to fluidic (necrotic/non-enhancing tumor core II, magenta) and solid (necrotic/non-enhancing tumor core I, red) components, within the gross tumor regions (right). Expected spatial correlations of the two subcomponents are maintained as the back-projection of the T1-T2-based classification of necrotic and solid tissue results in connected annotations (left)

**Fig. 8:**
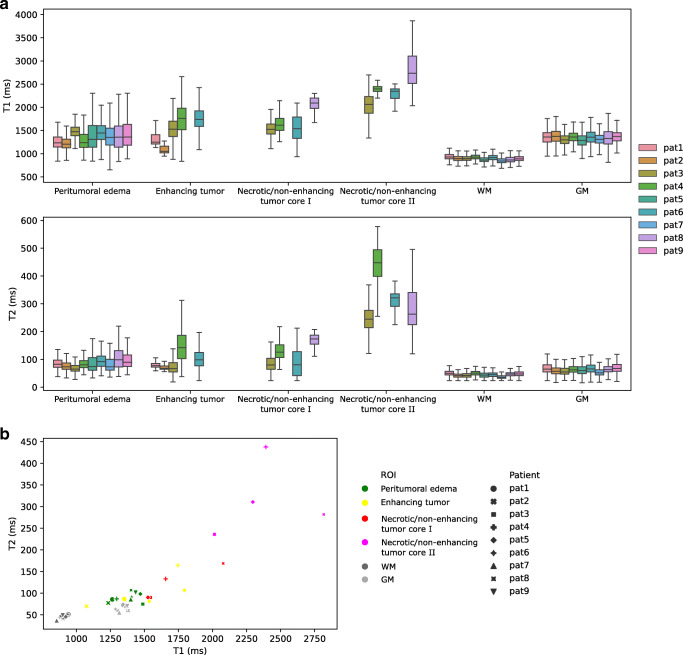
Quantitative ROI-based parameter analysis. Boxplots of the patient-wise T1 and T2 parameter spaces (**a**) and a scatter plot of the respective mean T1 and T2 values (**b**) indicate increased T1 and T2 values in diseased, tumorous regions compared to healthy, contralateral WM and GM regions with high inter-subject consistency and small variance. Outliers in the boxplots are omitted for clarity

Figure 7 illustrates exemplary results for patient case 4 and 6 that we obtained from the Gaussian mixture model, when trained on all voxels in the patients’ dataset labeled as necrotic/non-enhancing tumor tissue. The two voxel types, i.e., necrotic/non-enhancing tumor I (red) and necrotic/non-enhancing tumor core II (magenta) that are identified in the T1-T2 parameter space were projected back into the anatomical context to complement the manual ROI segmentation. Figure 8 and Table 3 quantitatively summarize the ROI-based analysis of tumor substructures. Quantitative T1 and T2 mapping results obtained in a healthy volunteer are reported in Table 4.

**Table 3:** Quantitative summary of T1 and T2 values (mean, standard deviation) of the tumor ROIs and contralateral WM and GM regions

ROI	T1 (ms)		T2 (ms)	
	Mean	Std	Mean	Std
Enhancing tumor	1685	340	111	70
Necrotic/non-enhancing tumor core I	1550	195	92	38
Necrotic/non-enhancing tumor core II	2188	375	297	111
Peritumoral edema	1369	264	91	35
WM	903	76	46	11
GM	1353	184	66	24

## Discussion

This study aimed to evaluate the feasibility of 3D quantitative transient-state imaging (QTI) for clinical imaging of glioma patients. First, we demonstrated a feasibility analysis of QTI-based, fully 3D multiparametric MRI for integration into state-of-the-art clinical routine brain tumor protocols with strict requirements regarding acquisition times and robustness. Second, we showed that 3D QTI offers comprehensive characterization of both healthy and diseased tissue in a variety of brain tumor patients. Despite the heterogeneity of the patient cohort, this approach captures tissue heterogeneity in tumor substructures based on quantifiable T1 and T2 parameters.

### Initial clinical experience with 3D QTI

#### Multiparametric mapping

**Fig. 9:**
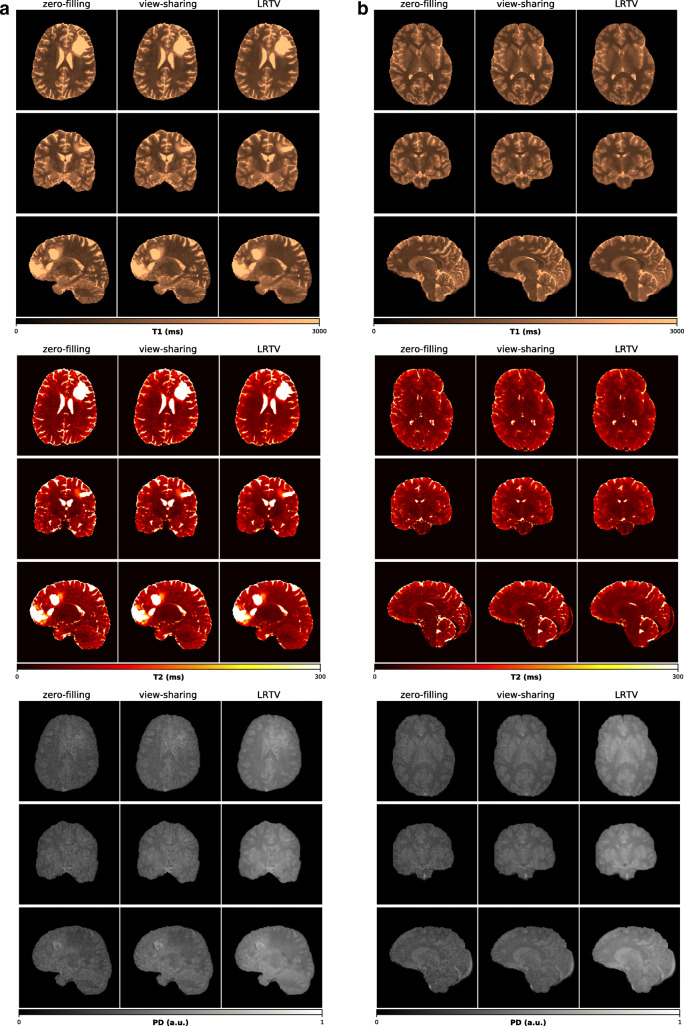
Parametric maps of T1, T2, and PD obtained with zero-filling, k-space weighted view-sharing, and LRTV reconstructions and subsequent dictionary matching for a representative patient case (**a**) and a healthy volunteer (**b**). Parameter estimation is consistent across all reconstruction approaches. View-sharing reconstruction provides better image quality in the quantitative maps than zero-filling with best reduction of undersampling artifacts obtained with LRTV reconstruction

Initial experience with 3D QTI in glioma patients demonstrated fully quantitative, multiparametric MR mapping with high isotropic resolution and an acquisition time of 6:25 min that make it feasible for use under tight clinical time constraints. We observed that parameter quantification is consistent across the different reconstruction approaches provided by the 3D QTI pipeline, i.e., zero-filling, view-sharing, and LRTV methods (Fig. 9). This is in correspondence with previous study results [18, 21]. View-sharing and LRTV reconstruction can improve spatial consistency in the reconstructed SVD image series compared to naïve zero-filling as reflected in an increased image quality of the inferred parameter maps. The iterative LRTV reconstruction with joint spatio-temporal regularization achieves best suppression of aliasing artifacts. It provides best image quality and maintains clinically important tissue changes and critical tissue interfaces within tumor and peritumoral regions. That is, complementing the fast 3D QTI acquisition with a compressed sensing reconstruction with joint spatio-temporal regularization has demonstrated convincing capacities to suppress aliasing artifacts, producing high-quality parametric maps. In 3D QTI, acquisition and reconstruction are well aligned, allowing to successfully mitigate inherent practical concerns of spiral sampling such as gradient imperfections or spiral artifacts due to a massively undersampled k-space. As such, we take advantage of the high scanning efficiency of spiral trajectories and use it in clinical routine imaging, as an alternative to the prevalent Cartesian readout schemes. Based on the initial results presented here, we are confident that we can further advance 3D QTI, e.g., to smaller voxel sizes or faster scanning times.

**Fig. 10:**
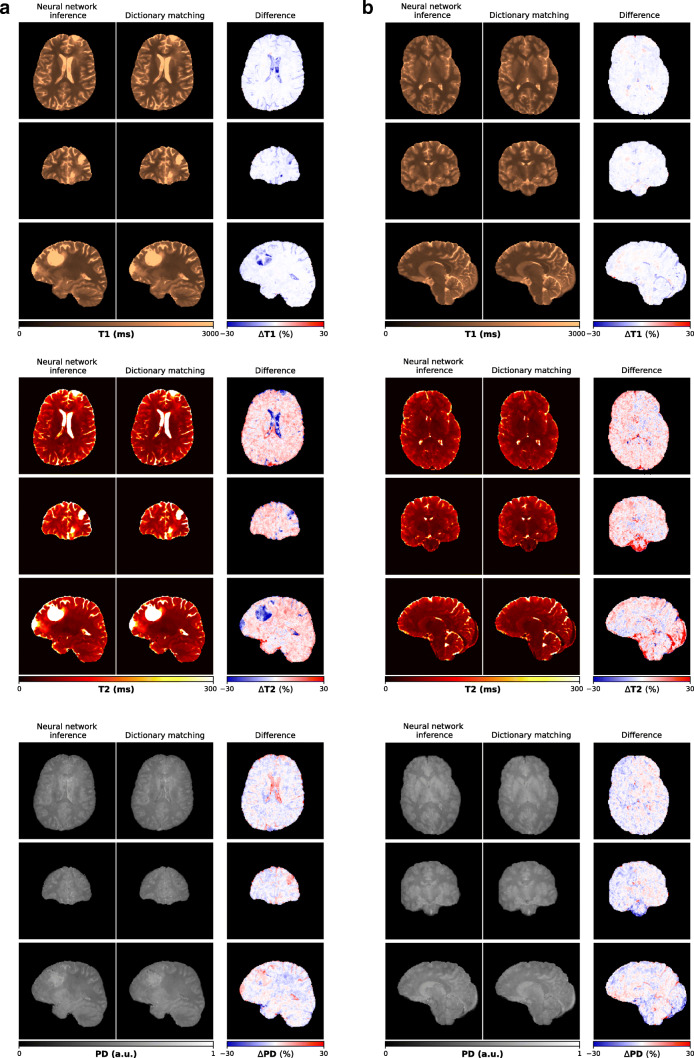
Neural network-based parameter estimation for a representative patient case (top) and a healthy volunteer (bottom). T1, T2, and PD maps obtained from voxel-wise neural network inference are consistent with dictionary matching results. The parameter maps are computed from SVD-compressed image-series after LRTV reconstruction

**Fig. 11:**
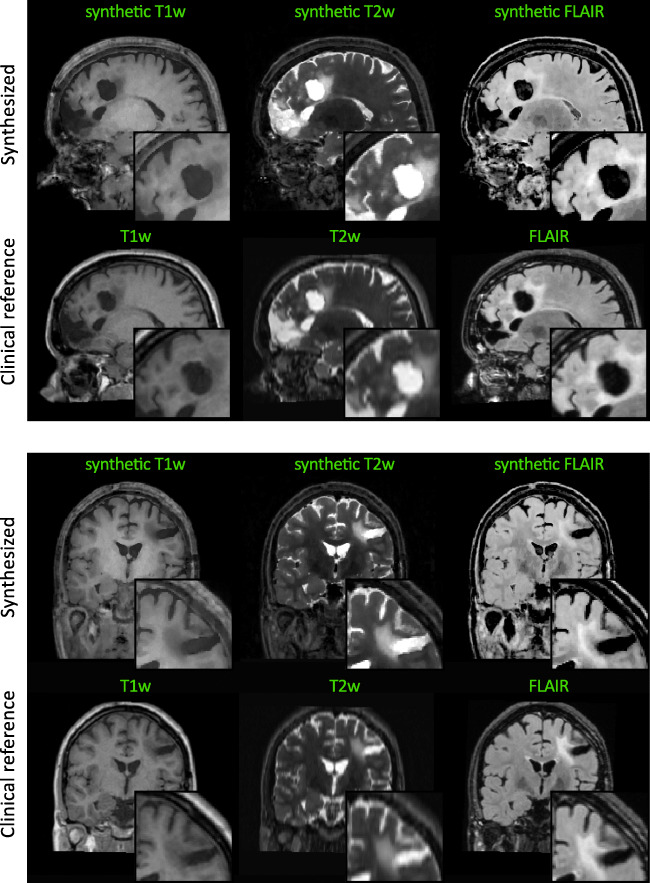
Contrast-weighted image synthesis for a representative patient case. Sagittal and coronal views in correspondence to the axial views in Fig. 3 are shown

**Fig. 12:**
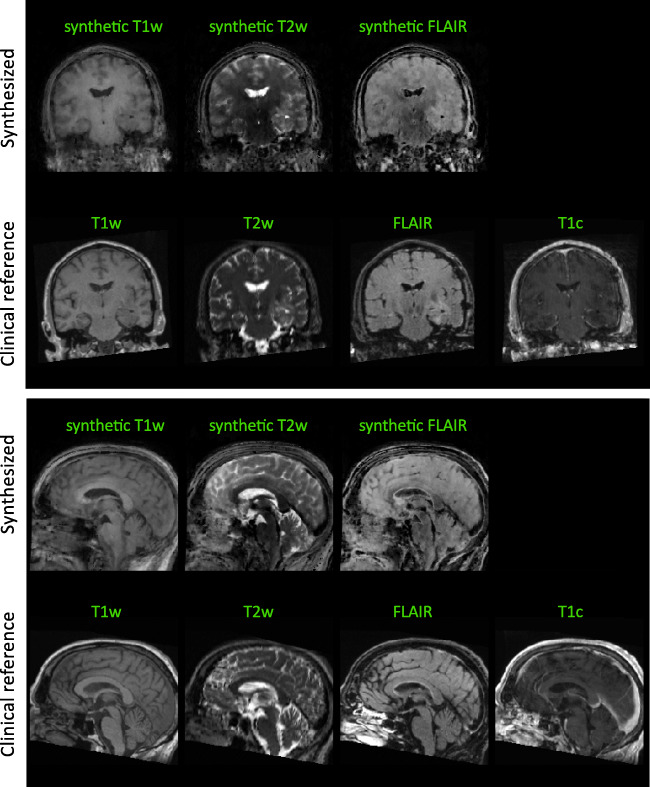
Sensitivity to rigid head motion. Sagittal and coronal views in correspondence to the axial views in Fig. 4 are shown

Comparison of neural network-based inference and dictionary matching (Fig. 10) showed that both approaches produce T1, T2, and PD maps that are largely consistent in terms of quantification accuracy and image quality as previously shown by Gómez et al. [18]. As such, the neural network provides high-resolution maps with quantification accuracy and image quality comparable to dictionary matching. This is observed for healthy tissue, i.e., of the volunteer scan and normal-appearing tissue regions in glioma patients, as well as in tumor regions with alterations of the tissue microstructure.

With the combination of the CS-based LRTV reconstruction and the neural network-based parameter inference, we therefore present a memory-efficient, dictionary-free reconstruction pipeline.

#### Synthetic MRI

Given the premise of an as-short-as-possible imaging protocol, we have shown that contrast-weighted image synthesis based on the multiparametric 3D QTI output can produce fully 3D, high-quality, and clinically relevant qualitative information without prolonging the scan session (Fig. 3). As such, it offers an attractive feature with the potential to replace conventional contrast-weighted acquisitions, including T1-weighted, T2-weighted, and FLAIR contrasts, to potentially reduce the required scan times of routine brain imaging protocols (Table 2). Note that the synthetic contrast-weighted MRIs are naturally obtained in the same image space. That is, expensive processing, i.e., co-registration and resampling of image volumes, which is generally a key requirement in multimodal studies in order to homogenize the individual datasets, becomes redundant. So far, image synthesis based on T1, T2, and PD estimates is confined to native, i.e., pre-contrast image contrasts, and can therefore not replace Gd-enhanced acquisitions yet. In light of the ongoing research efforts to reduce the use of contrast agents to an absolute minimum, there have been initial studies suggesting that T1-relaxometry can potentially provide equivalent insights into tissue characteristics as qualitative post-contrast information [33]. However, based on our results, we cannot draw such conclusions solely based on native T1 and T2 parameters, i.e., without the inclusion of diffusion information which is also part of recent research works [34–37].

#### Motion sensitivity

Subject motion is known to affect the quality of the reconstructed transient-state image time-series, which then propagates to the estimation of tissue parameters [38, 39]. 3D QTI was found to be tolerant to marginal head movements so that we achieved image qualities of the parametric maps comparable to qualitative, state-of-the-art protocols. We attribute this to the fast acquisition based on undersampled spiral readouts, which repeatedly sample the k-space center and are therefore more robust to motion already in the first place. This is particularly advantageous for severely diseased patients with difficulties to lie still during lengthy scanning sessions. However, initial experience also revealed that more pronounced patient motion can degrade the image quality of reconstructed image time-series and biases estimated parameter maps (Fig. 4). The axial, sagittal, and coronal views showcase that depending on the actual motion pattern, image quality is not homogeneously degraded in all spatial directions. For instance, despite the motion-caused image blurring, image quality in the sagittal direction of the motion-affected synthetic T2-weighted image is still comparable to the clinical T2-weighted PROPELLER acquisition with a native slice thickness of 3 mm and thus lower spatial resolution in this direction. Further, from the image artifacts that are apparent in the post-contrast T1-weighted MRI, it becomes clear that patient motion is also a major challenge in state-of-the-art conventional MRI. While patient motion manifests as diffuse image blurring in case of the spiral 3D QTI readout, we observe typical ghosting artifacts for the Cartesian readout scheme of the clinical post-contrast T1-weighted scan due to the unique frequency- and phase-encoding directions. Combining the 3D QTI framework with a motion correction algorithm was previously shown to improve its robustness and can correct for patient motion [38]. Currently, this method can only correct for movements on a 7-s timescale, which could not sufficiently resolve the image degradation for the motion-affected patient case in our study.

In the same fashion as rigid head motion, the pulsating blood flow and the thereby induced pulsation of the cerebrospinal fluid (CSF) impact parameter estimation (Fig. 5). This is particularly seen in large vessels and in regions with high CSF pulsation, e.g., along the brainstem. Here, T2 values in the flowing blood are underestimated, which then reflects in lower signal intensities in the synthesized T2-weighted MRI compared to the clinical acquisition.

Given these findings, it is subject to our current and future work to also resolve motion on a faster scale, such as continuous rigid head motion, and to reduce sensitivity to physiological motion due to blood flow and/or brain pulsation.

#### Application to quantitative characterization of tumor substructures

#### Current state-of-the-art

Combination of advanced quantitative MR techniques together with contrast-weighted MRI has been shown to provide clinically relevant tissue information and is a key feature for precise tumor diagnostics: to date, state-of-the-art clinical routine MRI protocols, with acquisition times ranging from 20 to 60 min, generally comprise pre- and post-contrast T1-weighted, T2-weighted, and FLAIR sequences, which can be extended by T2*-weighted or susceptibility-weighted contrasts. Qualitative imaging is complemented by advanced quantitative MRI [40, 41] to capture tumor morphology and functionality, namely diffusion-weighted imaging and perfusion MRI [10]. Also, MR spectroscopy is used to improve brain tumor diagnostics and grading, although usually not as part of routine imaging. Diffusion tensor imaging and functional MRI provide essential information for surgery planning and guide tumor resection, as they inform the identification of tumor boundaries as well as localization of critical functional areas and neuron tracts.

Beyond the mentioned quantitative MRI schemes, which already made their way into clinical routine, several studies have shown that MR relaxometry can provide additional, clinically relevant information about critical tissue changes in gliomas that are not visible in contrast-weighted MRIs [42–44]. Among other findings, quantitative T1 and T2 mapping has been demonstrated to aid earlier detection of tumor progression compared to standard contrast-weighted MR imaging, due to an increase in T1 and T2 values in recurring glioblastoma [45, 46]. It has also been shown that detection of tissue changes in peritumoral regions can benefit from quantitative T1 and T2 mapping due to their earlier sensitivity compared to contrast-weighted MRI [47].

Although above mentioned methods have proven to offer critical measures for disease characterization and prognosis, they often require expensive off-line processing, involve case-specific tuning of sequence settings, or cannot meet the clinical time-constraints that challenge their standard use in clinical brain tumor imaging protocols.

A variety of advanced multiparametric relaxometry techniques [12, 13, 48], including the pioneering work on MR fingerprinting (MRF) [14, 28], have been shown to offer fast, robust, and user-friendly quantitative MRI to be easily integrated into radiological practice. Its attractiveness in terms of scan times together with its high degree of repeatability [32, 49, 50] makes these techniques attractive candidates for providing relaxometry-based biomarkers in day-to-day brain tumor diagnosis.

Despite the increasing number of recent works that focus on transient-state-based relaxometry techniques, there is only a modest number of studies to investigate their feasibility in disease-specific setups, such as glioma imaging [51–53]. These works have for example demonstrated the feasibility of MRF for brain tumor characterization. While these studies are based on 2D acquisitions with discrete slices placed in the tumor regions, our results in a patient cohort with a realistic, heterogenous clinical picture in terms of disease and treatment histories (Table 1, Fig. 6) demonstrate a fully 3D whole-brain quantitative analysis of tumor information captured in T1 and T2 estimates.

#### Qualitative comparison of the tumor cases

Our feasibility study of a variety of glioma patients showed that 3D QTI can be viable for tissue characterization and discrimination. Qualitatively, a ROI-based analysis of voxel-wise T1 and T2 relationships revealed homogenous distributions for peritumoral edema and enhancing tumor regions. In case of necrotic/non-enhancing tumor tissue, T1-T2 parameter spaces seemed to be composed of two classes, which we attributed to fluidic and solid tissue components within the gross tumor. Building on the clinical expert annotation, we aimed to gain more insight into necrotic/non-enhancing tumor tissue and explored whether a Gaussian mixture model can disentangle these two voxel classes (Fig. 7). Linking the thereby identified tissue subclasses to the anatomical space reveal spatially well-connected annotations. In clinical routine tumor annotation, solid and fluidic parts within the necrotic/non-enhancing tumor regions are generally not differentiated and comprised in one overall ROI. That is why we do not have a reference annotation to compare the results of the Gaussian mixture model with. Nevertheless, we believe that our explorative analysis is an illustrative example of how quantitative, multiparametric measures of the underlying relaxation times can complement tumor annotations that are generally based on qualitative, visual abnormalities in contrast-weighted MR image data.

#### Quantitative analysis of tumor substructures

Quantitative analysis (Fig. 8 and Table 3) substantiated and complemented these qualitative findings. We observed that tumor tissue, i.e., enhancing tumor, necrotic/non-enhancing tumor core I + II, as well as peritumoral edema, exhibit higher T1 and T2 values than healthy contralateral WM, as qualitatively suggested from Fig. 6. These findings are in line with initial clinical outcomes of MR fingerprinting [51], supporting the potential of 3D QTI as an image-based biomarker for glioma diagnosis.

Figure 8 also suggests well-defined parameter spaces for healthy WM and GM regions among the patient cohort with only small inter-subject variations. Also, T1 and T2 mapping in non-diseased tissue was found to agree well with our results for healthy volunteer data, as suggested by Table 3 and Table 4, and is consistent with previously reported values [18, 38, 54, 55]. Furthermore, mean T1 and T2 values of the two gross tumor subclasses are well distinguished. That is, mean T1 and T2 values for necrotic/non-enhancing tumor core II, which we attributed to the fluidic subcomponent, are constantly higher than for necrotic tumor/non-enhancing core I. This finding agrees with the fact that T1 and T2 relaxation times are sensitive to tissue composition and microstructure [56, 57].

Overall, T1 and T2 values suggest a clear distinction between peritumoral edema and contralateral WM (Fig. 8b). Also, mean T2 values in peritumoral edema were higher than for contralateral GM in all patients. Mean T1 values in peritumoral edema were lower compared to necrotic/non-enhancing tumor regions. For mean T2 values, this is only observed in case of patients 4 and 8. As observed from Fig. 8b, enhancing tumor overlays with peritumoral edema and necrotic/non-enhancing tumor core I in the T1-T2 space. Enhancing tumor is segmented as those regions within the gross tumor that exhibit positive Gd enhancement in the Gd-enhanced T1-weighted MRI. Native pre-contrast T1 maps hence do not necessarily capture this differentiation.

Due to the observed inter-subject consistency and stability of T1 and T2 values of healthy and diseased tissue, which is in line with previously performed repeatability studies [32, 58, 59], we believe that not only diagnosis but also treatment planning and monitoring as well as prognostic, longitudinal assessment might benefit from an integration of 3D QTI into standard clinical imaging.

While conventional contrast-weighted MRI can also capture tissue heterogeneity in tumor texture, it always represents weighted information of a combination of relaxation and tissue parameters. Also, qualitative MRI information is known to depend on the actual scanner settings, which can vary from day to day due to different pre-scan conditions or gain tunings, hampering longitudinal or inter-subject comparisons.

Despite the mentioned benefits of fast multiparametric mapping techniques, we emphasize that both quantitative and qualitative MRI are generally limited to macroscopic voxel sizes. Resulting partial volume effects, e.g., at the border of distinct tissue types, are known to mask the underlying cellular tissue characteristics, only providing an effective voxel value of the respective biomarker. However, aiming for high isotropic resolution with sufficient SNR, as in case of 3D QTI, minimizes partial volume effects in the first place. Comparing the synthetic T2-weighted MRI generated from the 3D QTI scan with the clinical 2D reference with higher slice thickness in Fig. 3 illustrates this resolution benefit, e.g., for tumor delineation.

Nevertheless, with 3D QTI producing reliable, reproducible, quantitative image data, it may be a valuable tool for the harmonization of imaging data with the opportunity to make multi-timepoint, multi-subject, multi-vendor, and multi-center studies easier. As such, it can offer a rich set of standardized, comprehensive image data with the potential to also advance methodological developments along the large spectrum of AI-based decision support, i.e., segmentation algorithms or tumor growth modeling.

### Limitations

A clear limitation to this study is the rather small sample size. We aimed to present an initial clinical case study demonstrating the feasibility of 3D QTI for integration into routine glioma imaging protocols. As such, the rather small but heterogenous study cohort, comprising both pre- and post-surgery treatment stages as well as different tumor grades, allowed us to nevertheless cover the bandwidth of patient cases in clinical radiological practice.

### Outlook

Given our initial clinical results, we would like to employ 3D QTI also for longitudinal follow-up of glioma patients to evaluate its potential for monitoring and quantifying treatment response in individual subjects to support individualized therapy decisions with the aim of personalized medicine. We also plan to investigate how deep learning-based segmentation and interpretation of images [60, 61] could benefit from complementing or even replacing the to-date mainly qualitative data bases with quantitative imaging biomarkers, e.g., as provided by 3D QTI. Furthermore, we believe that extending the method to jointly encode relaxation and diffusion parameters as proposed in [37] with fully 3D, high isotropic resolution, and whole-brain coverage can further improve its attractiveness for integration into clinical glioma imaging and is hence subject of our current work. We also aim to further increase motion robustness of the transient-state encoding scheme. Building on the previously presented retrospective motion correction [38], we are optimistic that we can further develop this approach to account and correct for head motion on a faster time scale. We might also benefit from recent advances on prospective motion correction [62]. To reduce the sensitivity to non-rigid motion, such as blood flow and/or brain pulsation, in first place, it is subject to our future work to investigate potential refinements in the sequence design. The experience with 3D QTI in neuro applications also motivates us to target potential diagnostic scenarios outside the brain as well as in combination with contrast enhancement [63, 64].

## Conclusion

3D QTI demonstrated to reliably identify tissue and hence tumor heterogeneity that is captured in T1 and T2 relaxation times under tight clinical time constraints. As such, it offers comprehensive tissue assessment of tumor substructures with the potential to improve disease characterization in brain tumor patients. This is essential to find the optimal treatment strategy and to monitor treatment response along the course of disease.

## Data Availability

The data presented in this study are available on reasonable request from the corresponding author. The data are not publicly available due to restrictions on the use of confidential data in the written consent provided by participants.

## Appendix

**Table 4:** Quantitative summary of T1 and T2 values (mean, standard deviation) of WM and GM regions in a healthy volunteer

	T1 (ms)		T2 (ms)	
ROI	Mean	Std	Mean	Std
GM	1331	137	66	12
WM	871	77	45	7
